# Preparation, Purification and Regioselective Functionalization of Protoescigenin—The Main Aglycone of Escin Complex

**DOI:** 10.3390/molecules18044389

**Published:** 2013-04-15

**Authors:** Mariusz M. Gruza, Kamil Jatczak, Bogdan Zagrodzki, Marta Łaszcz, Katarzyna Koziak, Maura Malińska, Piotr Cmoch, Tomasz Giller, Oliwia Zegrocka-Stendel, Krzysztof Woźniak, Grzegorz Grynkiewicz

**Affiliations:** 1 Pharmaceutical Research Institute, Rydygiera 8, Warszawa 01-793, Poland; E-Mails: m.gruza@ifarm.eu (M.M.G.); k.jatczak@ifarm.eu (K.J.); b.zagrodzki@ifarm.eu (B.Z.); m.laszcz@ifarm.waw.pl (M.Ł.); piotr.cmoch@icho.edu.pl (P.C.); t.giller@ifarm.eu (T.G); 2 Department of Immunology, Biochemistry and Nutrition, Medical University of Warsaw, Żwirki i Wigury 61, Warszawa 02-091, Poland; E-Mails: katarzyna.koziak@wum.edu.pl (K.K.); ostendel@wum.edu.pl (O.Z.-S.); 3 Department of Chemistry, Warsaw University, Pasteura 1, Warszawa 02-093, Poland; E-Mails: mauramalinka@gmail.com (M.M.); kwozniak@chem.uw.edu.pl (K.W.); 4 Institute of Organic Chemistry Polish Academy of Sciences, Kasprzaka 44/52, Warszawa 01-224, Poland

**Keywords:** escin, protoescigenin hydrates, protoescigenin diacetonide, polymorphism, X-ray crystallography

## Abstract

A two-step chemical process for controlled degradation of escin, affording a mixture of olean-12-ene sapogenins, was elaborated and scaled up. The main component of the mixture—protoescigenin—was isolated and purified, in the form of its corresponding monohydrate, without resource to chromatographic methods. This material was further converted into the high purity 3,24;16,22-di-*O,O*-isopropylidene derivative in a validated large scale laboratory process.

## 1. Introduction

**Scheme 1:**
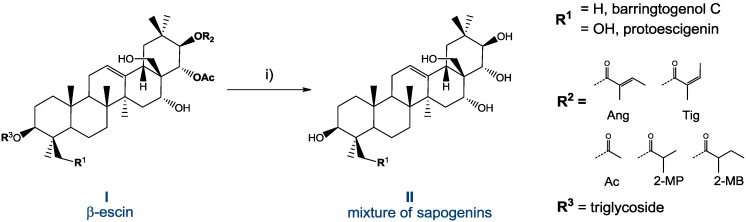
Conversion of escin (mixture of saponins) into their aglycons (only protoescigenin and barringtogenol C are shown).

Escin (aescin), a saponin complex from horse chestnut seeds (*Aesculus hippocastanum* L.), constitutes a traditional herbal drug which enjoys a good clinical reputation as a treatment for chronic venous insufficiency and capillary blood vessel leakage [1,2,3,4]. Although considerable effort of many phytochemical groups has been put into studies of the structure of the individual saponin constituents [5,6,7], pharmaceutical preparations of escin usually suffer from poor specifications and the biological activities of individual members of the multicomponent mixture are practically unknown. Since preparative separation of escin constituents presents a paramount challenge, even for advanced techniques, there is not much hope for availability of single escins, some of which might be characterized by much higher efficacy than the saponin mixtures presently used. In many cases biologically active natural products are transformed through semi-synthesis on their way to modern drugs [8,9] and saponins bearing triterpenoid aglycons are no exception [10,11]. For escin, this avenue may prove unmanageable when the native complex is considered, therefore we propose a more radical approach to exploitation of the complex anew, in which the saponin mixture is perceived as a source of a hitherto unavailable raw material—protoescigenin (**1**, Scheme 1).

*Reagents and Conditions*: (i) (a) H_2_SO_4_, MeOH, reflux, (b) KOH, H_2_O.

## 2. Results and Discussion

### 2.1. General

Plant secondary metabolites are considered invaluable sources of structural diversity—a feature much sought after in medicinal chemistry and drug development programs. Although a great many modern medicines stem from natural products, their early development is frequently hampered by lack of active substance because the initial isolation procedure is inefficient, while technical process elaboration securing API supply of appropriate quality and in quantities suitable for pre-clinical and clinical studies, usually lags behind. In our studies, which are inspired by escin saponin complex as a useful drug inherited from ethnopharmacological tradition, but not compatible with modern medicine requirements, we decided to address the problem of substance availability and specified quality from the very beginning. Since separation of the native saponin mixture as a large scale process seemed unattainable, a semi-synthesis strategy was designed, based on pentacyclic triterpene sapogenins as principal intermediates. Protoescigenin (**1**)—the main aglycon of escin saponins—was picked as the first candidate molecule, because although known as chemical entity from the classical period of triterpene saponin exploration [12,13] and more recently confirmed as 3β,16α,21β,22α,24,28-hydroxyolean-12-ene by modern analytical and spectral tools [14], it is not commercially available and its chemistry is practically unexplored.

### 2.2. Preparation and Isolation of **1**

Based on results of our preliminary experiments, which unsucessfully attempted a single step process for accommodation of two types of hydrolytic cleavage—de-glycosylation and de-esterification—a two-step procedure (sequential one-pot reaction: H^+^ followed by OH^−^ conditions) was adopted, which is outlined in Scheme 1.

**Scheme 2:**
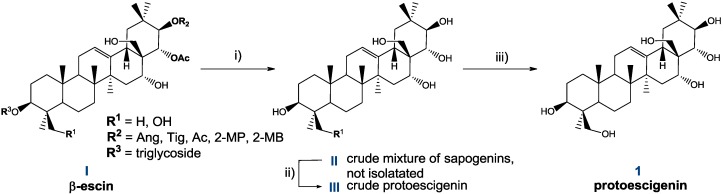
Original method of preparation of protoescigenin (**1**) from β-escin (**I**).

The mixture of sapogenins **II**, obtained by two-step hydrolysis, contains protoescigenin, barringtogenol C, barringtogenol D, escigenin and other triterpenoid components. Depending on handling, **II** can be obtained as a long lasting suspension, solids of different appearance or a resinous mass, containing up to 60% of protoescigenin (**1**). Isolation of **1** requires specific treatment with a mixture of solvents which is outlined on Scheme 2. In the initial laboratory experiments, this mixture was purified by column chromatography and pooled fractions of the main constituent were subjected to crystallization from methanol to afford pure **1**.

*Reagents and Conditions*: (**i**) (a) H_2_SO_4_, MeOH, reflux, (b) KOH, H_2_O; (**ii**) MTBE, MeOH, H_2_O; (**iii**) (a) *i*-PrOH, H_2_O, (b) (*i*-PrOH, cyclohexane.

The main focus, however, was on a method of isolation of **1** without using chromatographic protocols. We have checked standard methods of similar compounds separation from mixtures, e.g., filtration, extractions (liquid–liquid, solid-liquid), precipitation, crystallization and ultrafiltration through commercially available membranes (Paal, 600 Da), but none of these gave satisfactory results.

**Scheme 3:**
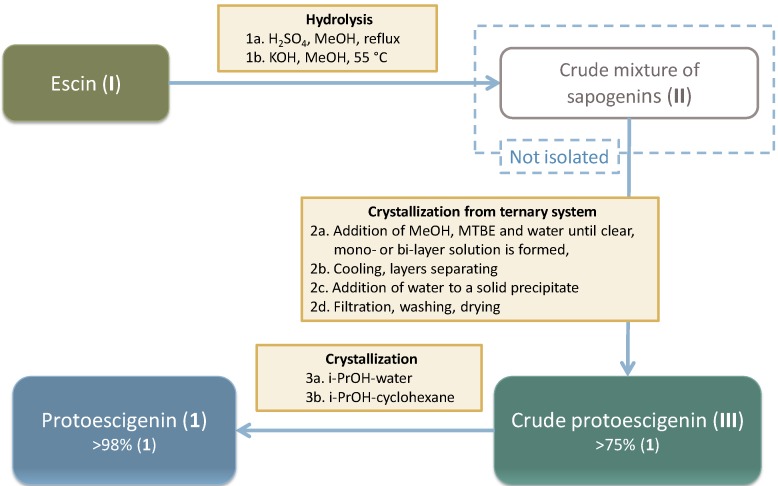
Technical scheme presenting flow of materials in the escin hydrolysis process, followed by isolation and purification of protoescigenin.

However, dissolution of the mixture **II** in three solvents (mixture of an alcohol, ether and water), followed by addition of water, allowed us to obtain a crystalline precipitate **III** with considerably improved content of **1** (Scheme 2, Scheme 3). Solid **III** typically contains from 70 to over 90% (HPLC) of **1**, barringtogenol C (the main impurity, from 3 to over 20%), and other substances in low quantities. The method of preparation of **III** has been optimized, and then scaled up. Eventually, the problematic step of isolation of mixture **II** was omitted, and a two-step hydrolysis and isolation of **III** were conducted sequentially in a one-pot process. Best results were obtained by carrying out: 1) the acid hydrolysis in MeOH in the presence of conc. H_2_SO_4_, followed by 2) basic hydrolysis with NaOH, and 3) the dissolution of the resulting slurry in a mixture of MeOH, MTBE and water. The phases formed are separated, and addition of water to the organic phase allowed us to obtain a precipitate **III**.

The solid **III** was subjected to crystallizations from *i*-PrOH—cyclohexane and *i*-PrOH—water, from which protoescigenin monohydrate (**1·H_2_O**) was obtained as a solid of consistent purity over 98% (HPLC) [15].

### 2.3. Characteristics of Solid Forms of **1**

**Figure 1:**
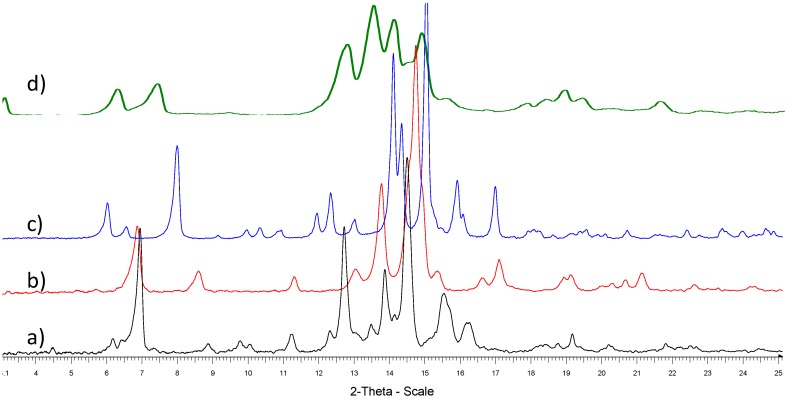
Powder diffractograms of protoescigenin hydrates forms: **II** (a), **III** (b), **IV** (c) and **VI** (d).

Protoescigenin was characterized by analytical methods, including thermal methods (m.p.; DSC; TGA), XRPD and single crystal X-ray diffraction, as well as water determination by KF. Purity of the samples was determined by HPLC and UPLC. Based on collected analytical data specifications for **1** and **2** as prospective pharmaceutical intermediates were defined. Since it is well established that the solid state characteristics play an important role in the qualification and certification of pharmaceutical materials, much effort has been invested in establishing the physicochemical characteristics of the obtained solids in an attempt to define and distinguish their polymorphic nature, first heralded by various contents of water in different samples of **1**, as determined by the KF method. Samples obtained from various isolation and purification procedures could be grouped as hydrates containing from 0.5 to 3.5 water molecules per triterpene molecule, each level of hydration exhibiting characteristic features in diffractograms (Figure 1, Table 1), although most of them clearly contained some amorphous matter resulting in poorly resolved peaks.

**Figure 2:**
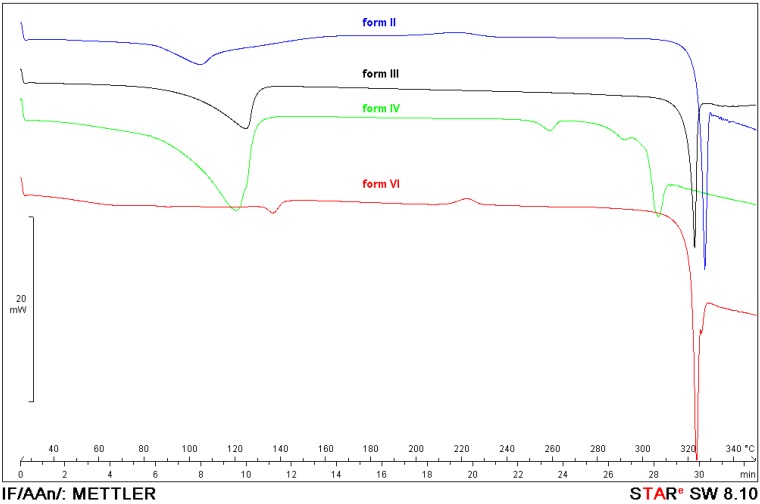
DSC curves of protoescigenin hydrate forms.

DSC curves of the protoescigenin hydrates forms exhibit a broad endothermic effect to about 160 °C as a result of water evaporation. In this temperature range TGA curves show significant mass losses. The comparison of mass losses and water content (obtained from KF titration) indicates that form **II** is a hemihydrate, form **III** is a monohydrate, form **IV** is a trisesquihydrate and form **VI** can be a hemihydrate or monohydrate. Melting temperatures are as follows: 326 °C (form **II**), 322 °C (form **III**), 306 °C (form **IV**), 322 °C (form **VI**). Decreasing lines of the DSC curves of the protoescigenin hydrates (Figure 2) visible after melting effects indicate an accompanying decomposition. The exothermic peak at about 220 °C visible in the DSC curves of forms **II** and **VI** comes from crystallization of an amorphous component. The DSC curve of form **IV** shows two additional endothermic effects at 260 and 290 °C that can be connected with melting of impurities.

**Table 1:** Characteristics of some hydrated forms of protoescigenin.

2θ [°]			
form II	form III	form IV	form VI
6.16	6.85	6.00	3.08
6.92	8.58	6.53	6.29
8.85	11.30	7.97	7.42
9.76	13.04	10.33	9.37
10.04	13.76	11.94	12.74
11.22	14.75	12.33	13.43
12.72	15.35	13.00	14.03
13.88	16.64	14.10	14.80
14.50	17.11	14.34	15.51
15.54	18.96	15.04	17.76
16.23	19.15	15.92	18.28
18.77	20.30	16.09	18.82
19.18	20.68	16.99	19.32
20.23	21.15	22.43	21.44
21.84	22.65	23.42	

**Figure 3:**
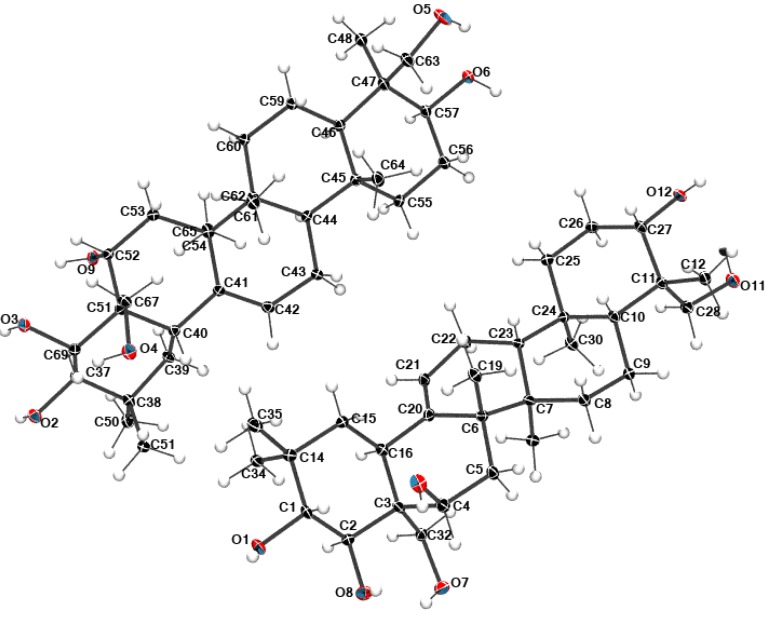
The atom labeling scheme of the molecular structure of **1**. Thermal displacement ellipsoids are drawn at the 50% probability level.

Some highly hydrated samples tend to produce regular crystals upon treatment with single solvents (lower alcohols) or binary solvent systems (e.g., *i*-PrOH–cyclohexane), facilitating X-ray diffraction measurement, which gave clear picture of elementary cell (with two triterpene molecules), accompanied by seven water molecules exhibiting a severe disorder. For clarity only triterpene molecules are shown in the Figure 3.

### 2.4. Preparation and Isolation of **2**

**Scheme 4:**
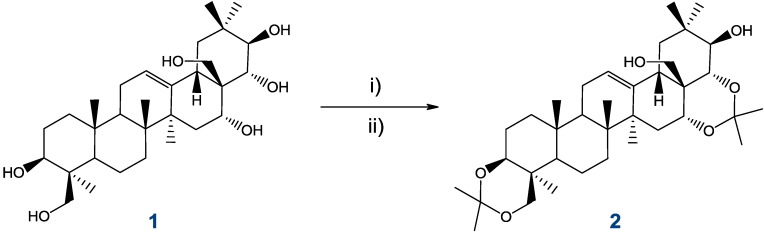
Selective isopropylidation of protoescigenin.

Compound **1** is olean 12-ene derivative, containing six hydroxyl groups: two primary (24 and 28) and four secondary (3β, 16α, 21β, 22α). Attempts at selective functionalization of the hexaol, even with use of bulky reagents routinely used for differentiation of hydroxyl functions in polyhydroxylated substrates, resulted in a complex mixture of products. Partial protection of **1** was achieved after many trials, in a ketalization procedure which facilitated isolation of a single product by filtration. Conversion of **1** into its diacetonide **2** [12,16] is illustrated in Scheme 4.

*Reagents and Conditions:* (**i**) 2,2-DMP, p-TSA, acetone, 80–85% (purity > 94%, HPLC); (**ii**) MTBE, reflux (purity > 99%, HPLC).

**Figure 4:**
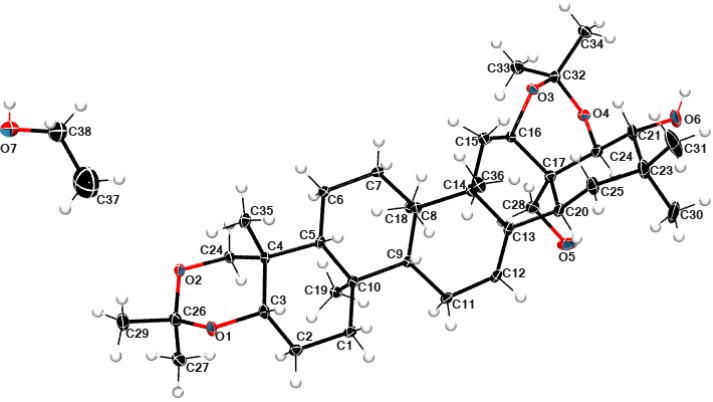
The atomic labeling of the molecular structure **2** with ethanol solvent molecule. Thermal displacement ellipsoids are drawn at the 50% probability level.

Primary filtrate of the reaction mixture was purified by maceration in MTBE to over 98% (HPLC) level in 50 g batches [17]. Apart from routine spectral characteristics, crystals of acetonide **2** ethanol solvate was subjected to single crystal diffraction, which provided additional proof of its molecular structure (Figure 4).

### 2.5. Structural Data

Compound **1** crystallizes in P2_1_2_1_2_1_ orthorhombic space group with two molecules of hydroxylated olean 12-ene derivative in the asymmetric part of the unit cell. After structure solution, it was found that ca. 13% of the total cell volume was filled in with seven disordered molecules of water, which could not be modeled in terms of atomic sites (for details see the Experimental Section). Di-*O*-isopropylidene protoescigenin **2** crystallizes in the monoclinic P2_1_ space group with one molecule in the asymmetric unit and one ethanol moiety. The numbering scheme of atoms with atomic displacement parameters calculated at the 50% probability level for **1** and **2** are shown in Figure 3, Figure 4, respectively.

**Figure 5:**
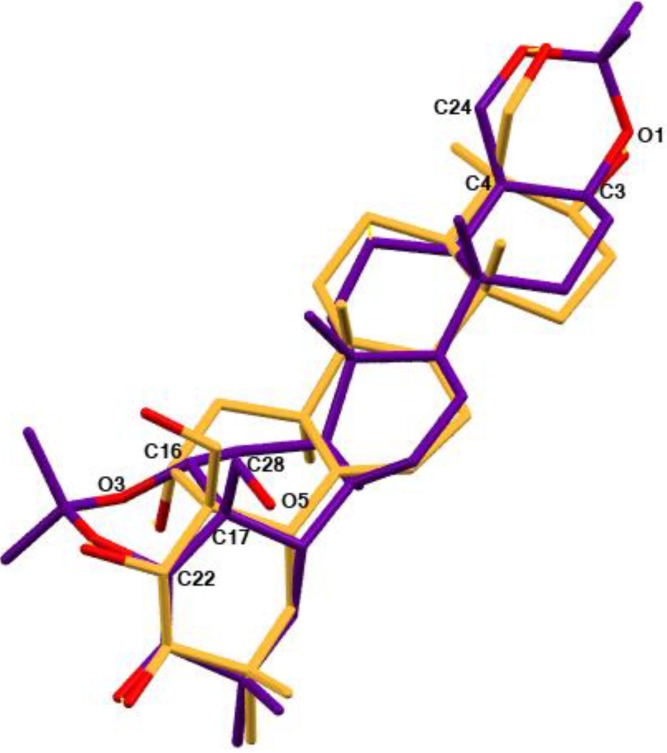
Overlay of structure **1** (yellow) and **2** (purple). For the clarity of the picture only selected atom labels for structure **2** have been shown.

The diacetonide analog **2** with two additional rings has a similar atom orientation as the original compound **1**. A structural overlay for molecules **1** and **2** is shown in Figure 5. The molecules have a typical geometry of the condensed rings common for all protoescigenin analogs. The most significant difference is the orientation of the hydroxyl group O5-H5, and in consequence different values of the C2-C3-C32-O7 and C22-C17-C28-O5 torsion angles which are equal to 64.9(3)° and −65.8(2)° for 1 and 2, respectively. Additionally, the diacetonide substitution to protect the hydroxyl group results in the modification of the C2-C3-C4-O10 torsion angle from −55.5(2)° to −12.3(3)° (C22-C17-C16-O3). Nonetheless, the X-ray crystal structure studies confirm that both structures adopt the same conformation. The crystal and molecular structures of different diacetonide analogs of **1** was previously reported [18].

**Figure 6:**
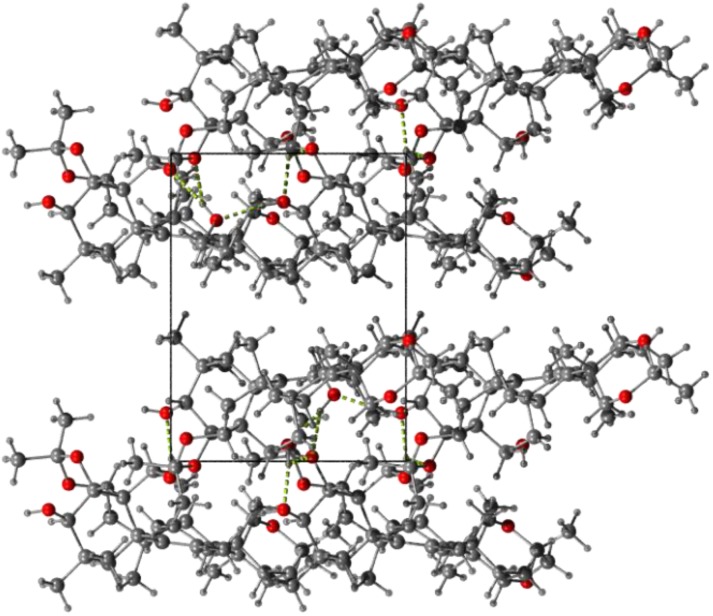
Crystal packing of molecules of **2**—view along the Z axis. The O-H…O hydrogen bond is drawn as green dashed lines.

The packing of molecules reveals a network of hydrogen bonds which form a ribbon-like motif along the Y directions. Two molecules of **2** form chain motifs linked via the O5—H5A·O6*_x, y+1/2, -z+2_* hydrogen bonds which associate further via O6—H6…O7*_x, y-1, z+1_* and O7—H7…O5*_x, y, z-1_* hydrogen bonds with the solvent molecules thus creating a ribbon-like pattern. The donor-acceptor distances for the above bonds are: 2.725(3) Å, 2.629(4) Å and 2.706(3) Å, respectively. Secondly, weak C-H..O and H…H interactions also occur between the hydrophobic part of the structure studied, forming the layer structure (Figure 6) in the parallel direction to the previously mentioned arrangement.

## 3. Experimental

### 3.1. General

Escin (specified as β-escin, active pharmaceutical ingredient; COA JP 10061/K2) was provided by the manufacturer (Nobilus Ent., Jabłonna, Poland). Other materials, solvents and reagents were technical grade, released for use by Quality Control, Pharmaceutical Research Institute.

Differential scanning calorimetry (DSC) measurements were carried out by means of the DSC822 with IntraCooler (Mettler Toledo GmbH, Schwerzenbach, Switzerland). Specific rotation was calculated from an optical rotation measurement performed on a Perkin Elmer 341 Polarimeter (Perkin-Elmer, Santa Clara, CA, USA) at the wavelength of 589 nm (sodium lamp), at 20 °C. The ^1^H and ^13^C-NMR spectra were recorded at 298 K temperature in DMSO-d_6_ solutions with a Varian-NMR-vnmrs600 instrument (Varian, Palo Alto, CA, USA) equipped with a 600 MHz PFG Auto XID (^1^H/^15^N-^31^P 5 mm) indirect probehead. To assign the structures under consideration following 1D and 2D experiments were employed: 1H selective TOCSY, NOESY, 2D: COSY, ^1^H-^13^C gradient selected HSQC and HMBC for ^1^*J*(C-H) = 140 Hz and ^n^*J*(C-H) = 8 Hz, respectively. Standard experimental conditions and standard Varian programs (ChemPack 4.1) were used. The ^1^H and ^13^C-NMR chemical shifts are given relative to the TMS signal at 0.0 ppm, Concentration of solutions used for measurements was about 20–30 mg of compounds in 0.6 mL of solvent. Mass spectra (LR) were recorded on MS Applied Biosystems 3200 QTRAP (AB Sciex, Framingham, MA, USA) spectrometer *via* electrospray ionization (ESI-MS). Mass spectra (HR) were recorded on MaldiSYNAPT G2-S HDMS (Waters Corp., Milford, Taunton, MA, USA) spectrometer *via* electrospray ionization (ESI-MS). Related substances determination using High Performance Liquid Chromatography (HPLC) gradient method was carried out employing an UHPLC Dionex Ultimate 3000 system (Dionex, Sunnyvale, CA, USA), equipped with PDA detector, detection at λ = 200 nm, with 10 mM ammonium acetate and acetonitrile as mobile phase A and B, respectively. UHPLC conditions for compound **1**: Waters Acquity C18 BEH column (100 mm × 2.1 mm; 1.7 µm), gradient program: 20–100% of mobile phase B for 5 min, then in 100% B for 2 min and return to initial conditions plus column stabilization for 3 min; flow 0.5 mL/min, t_R_ of compound **1** ~3.80 min. HPLC conditions for compound **2**: Kinetex XB-C18 column (150 mm × 4.6 mm; 2.6 µm), gradient program: 20-100% of mobile phase B for 5 min, then in 100% B for 2 min and return to initial conditions plus column stabilization for 3 min, flow 1.5 mL/min, t_R_ of compound **2** ~6.20 min. Water content determination was done by Karl Fischer volumetric titration using the Methrohm 701 KF Titrino apparatus and the Methrohm 6.0338.100 electrode (Methrohm AG, Herisau, Switzerland). Thermogravimetric analyses (TGA) were carried out by means of the TGA/SDTA851e (Mettler Toledo). X-ray powder diffraction (XRPD) studies were performed by means of the MiniFlex diffractometer (Rigaku Coporation, Tokyo, Japan) using CuKα radiation (λ = 1.54056 Å).

### 3.2. Preparation of Protoescigenin (**1**): Materials and Operations Shown in Scheme 2

To a solution of crystalline β-escin (3.0 kg) in methanol (11.85 kg) placed in glass reactor (50 L) equipped with mechanical stirrer, addition funnel, thermometer and heating/cooling device, a solution of sulphuric(VI) acid (95%, d = 1.84 kg/L, 1.20 kg) in methanol (1.20 kg) was added slowly, with stirring, then the reaction mixture was heated at 67–68 °C for 100 h. After cooling, a methanolic solution of sodium hydroxide (2.28 kg) was added and heating to 70–72 °C was continued for another hour. After switching off heating the mixture was stirred for the next two hours and then left at ambient temperature without stirring for 16–18 h.

**Figure 7:**
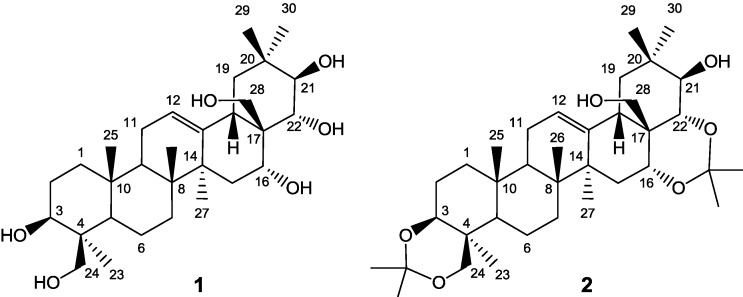
Structures of **1** and **2**.

The reaction mixture was transferred to a glass reactor (100 L), methanol (9.48 kg) was added and the mixture was heated to 50–55 °C. Next, *tert*-butylmethyl ether (TBME 24.5 kg) was added, maintaining the temperature, followed by water (27.7 kg). The reaction mixture was transferred to a separator (100 L) equipped with a stirrer, allowed to cool and the layers were separated leaving upper layer in the separator. The aqueous layer was extracted with *tert*-butylmethyl ether (11.15 kg) in a separate vessel and water (4.5 kg followed by 3.00 kg and 5.10 kg) was added to the combined organic layers, with stirring, which was continued for 16–18 h. Solids were separated by filtration, washed on the filter with two portions of diluted methanol (2.85 kg MeOH + 8.40 kg H_2_O). The filtrate was dried in air at 30–35 °C to constant mass, affording ca 0.45 kg of crude protoescigenin (ca. 75% by HPLC). This material was refined by treatment with isopropanol, cyclohexane and water in a rotary evaporator container (20 L), used as a heating under reflux device. First, crude protoescigenin (300.0 g) was dissolved in isopropanol (4.80 kg) at 55 °C and the hot solution was filtered to remove mechanical impurities. The filtrate was placed back on the rotary evaporator and heated to 85 °C (bath temperature), then water (3.30 kg) was sucked in (six portions separated by 10 min intervals). The mixture was allowed to cool with stirring and then it was left at ambient temperature for 16–20 h. Solids were collected by filtration and dried on air to a constant mass, then placed again on the rotary evaporator, dissolved in isopropanol at 85 °C and treated with cyclohexane (3.99 kg). After cooling and stirring at ambient temperature for 16–20 h, the precipitate was collected by filtration and washed on the filter with cyclohexane (0.80 kg) to afford protoescigenin monohydrate (197–209 g, purity >98% HPLC, solid form **III**). M.p. (DSC, for form **III**) 322 °C (dec.) (lit. [13,19,20]). **[α]^D^_20_** = +31.52 (*c* 1.0, EtOH, lit.[13,19,20]). MS C_30_H_50_O_6_, (ESI, positive): 507 [M+H]^+^, 524 [M+NH_4_]^+^, 529 [M+Na]^+^. Numbering of C-atoms is shown in Figure 7. 

^1^H-NMR (DMSO-d_6_), δ (ppm) lit. [14]: 5.19 (1H, m, H12), 4.96 (1H, d, *J* = 4.8 Hz, C3-OH), 4.43 (1H, dd, *J* = 4.4 and 5.8 Hz, C28-OH), 4.22 (1H, d, *J* = 4.4 Hz, C16-OH), 4.06 (1H, dd, *J* = 3.0 and 7.5 Hz, C24-OH), 4.03 (1H, m, H16), 3.97 (1H, d, *J* = 4.2 Hz, C21-OH), 3.82 (1H, m, H24), 3.81 (1H, d, *J* = 5.0 Hz, C22-OH), 3.79 (1H, dd, *J* = 4.2 and 9.6 Hz, H21), 3.60 (1H, dd, *J* = 5.0 and 9.6 Hz, H22), 3.27 (1H, dd, *J* = 7.6 and 11. 0 Hz, H24), 3.18 (1H, m, H3), 3.15 (1H, dd, *J* = 6.0 and 10.2 Hz, H28), 2.99 (1H, dd, *J* = 4.3 and 10.2 Hz, H28), 2.35 (1H, m, H19), 2.27 (1H, dd, *J* = 4.0 and 14.2 Hz, H18), 1.80 (2H, m, H11), 1.62–1.59 (2H, m, H15 and H2), 1.56–1.50 (4H, m, H6, H2, H1 and H9), 1.41 (1H, m, H7), 1.37 (1H, m, H6), 1.34 (3H, s, C14–CH_3_(27)), 1.25 (1H, m, H7), 1.19 (1H, dd, *J* = 2.3 and 14.8 Hz, H15), 1.08 (3H, s, C4–CH_3_(C23)), 0.94–0.90 (2H, m, H19 and H1), 0.87 (3H, s, C10-CH_3_(C25)), 0.84 (3H, s, C20-CH_3_(C29)), 0.81 (3H, s, C8-CH_3_(C26)), 0.80 (3H, s, C20-CH_3_(C30)), 0.75 (1H, dd, *J* = 1.8 and 12.0 Hz, H5). ^13^C-NMR (DMSO-d_6_), δ (ppm) lit. [14]: 143.1 (C13), 121.8 (C12), 78.6 (C3), 76.8 (C21), 74.0 (C22), 66.6 (C16), 65.2 (C28), 63.0 (C24), 55.4 (C5), 47.2 (C19), 46.2 (C9), 46.0 (C17), 42.1 (C4), 40.9 (C14), 39.5 (C18), 39.2 (C8), 38.2 (C1), 36.3 (C10), 35.3 (C20), 33.2 (C15), 32.8 (C7), 30.0 (CH_3_ 29), 27.2 (C2), 26.7 (CH_3_ 27), 23.2 (C11), 22.9 (CH_3_ 23), 18.8 (CH_3_ 30), 18.6 (C6), 16.3 (CH_3_ 26), 15.7 (CH_3_ 25).

### 3.3. Preparation of 3,24;16,22-di-O,O-isopropylideneprotoescigenin (**2**)

To suspension of protoescigenin monohydrate (**1**, 60.0 g) in acetone–2,2-dimethoxypropane mixture (300 mL each) stirred mechanically in a 1 L glass reactor, 4-toluenesulfonic acid monohydrate (0.39 g) was added and stirring was continued at ambient temperature (18–23 °C) for 18–20 h (TLC control; silica gel 60 F_254_ on aluminium sheets; hexane-ethyl acetate 1:1; cerium molybdate stain). Next, triethylamine (0.24 mL) was added and after 30 min. the solid precipitate was removed by filtration, washed with acetone and dried. Crude product was placed in a 2 L glass reactor equipped with a mechanical stirrer and reflux condenser and *tert*-butylmethyl ether (1.2 L) was added, followed by triethylamine (0.96 mL). The resulting mixture was stirred at reflux for 1.5 h, then cooled to 18 °C and stirring was continued for another hour. The precipitate was removed by filtration under reduced pressure and dried in air. The resulting white solid of **2** (55.0 g; 79.5% yield, purity >98% HPLC) had spectral data identical with the reference sample. M.p. (DSC) 288 °C (dec.) (lit. [12]). [α]^D^_20_ = +31.8° (*c* 1.0, THF). Numbering of C-atoms was shown in Figure 7. ^1^H-NMR (DMSO-d_6_), δ (ppm): 5.37 (1H, m, H12); 4.75 (1H, t, *J* = 4.8 Hz, C28-OH); 4.22 (1H, dd, *J* = 4.8 Hz), C21-OH); 3.92 (1H, d, *J* = 11.4 Hz, H24); 3.81 (1H, d, *J* = 9.0 Hz, H22); 3.66 (1H, dd, *J* = 4.8 and 9.0 Hz, H21); 3.41 (1H, m, H16); 3.37 (1H, dd, *J* = 4.8 and 9.0 Hz, H3); 3.31–3.14 (3H, m, H24 and 2 × H28); 2.0–1.8 (5H, m, H2, H11, H15, H19); 1.63 (1H, m, H2); 1.53–1.47 (4H, m, H1, H6, H9, H15); 1.45 (1H, m, H7); 1.39 (1H, m, H7); 1.34 (3H, s, (CH_3_)_2_C-); 1.33 (3H, s, (CH_3_)_2_C-); 1.30 (1H, d, *J* = 5.4 Hz, H6); 1.25 (6H, s, (CH_3_)_2_C-); 1.20 (1H, dd, *J* = 3.6 and 12.6 Hz, H19); 1.15 (3H, s, H27); 1.12 (3H, s, H23); 1.08 (3H, s, H25); 1.03 (1H, m, H1); 0.93 (3H, s, H29); 0.88 (1H, d, *J* = 12.0 Hz, H5); 0.78 (3H, s, H26); 0.77 (3H, s, H30). ^13^C-NMR (DMSO-d6), (ppm): 141.0 (C13); 121.7 (C12); 98.0 (C31); 97.8 (C34); 75.9 (C3); 75.7 (C21); 72.2 (C22); 68.3 (C16); 62.8 (C24); 62.3 (C28); 52.9 (C5); 46.8 (C9); 44.7 (C19); 43.1 (C17); 41.2 (C14); 40.3 (C18); 39.6 (C8); 36.9 (C4); 36.0 (C1); 35.8 (C10); 35.3 (C20); 35.0 (C15); 32.2 (C7); 30.4 (C29); 30.4 ((CH3)2C-); 27.9 (C27); 27.7 ((CH3)2C-), 25.7 (C23); 25.4 ((CH3)2C-); 24.4 (C2); 23.6 ((CH3)2C-), 22.7 (C11); 18.3 (C30); 18.2 (C26); 17.5 (C6); 16.7 (C25). HRMS (ESI): calcd for C_36_H_58_O_6_Na [M+Na]^+^: 609.4131 found: 609.4130.

### 3.4. Single Crystal X-ray Diffraction

The CCDC 922296 and 922247 entries contain the supplementary crystallographic data for **1** and **2**, respectively. These data can be obtained free of charge via www.ccdc.cam.ac.uk/conts/retrieving.htmL (or from the CCDC, 12 Union Road, Cambridge CB2 1EZ, UK; fax: +44-1223-336033; e-mail: deposit@ccdc.cam.ac.uk).

After the structure of **1** was solved, it was found that 13% of the total cell volume was filled with disordered solvent molecules, which could not be modelled in terms of atomic sites. From this point on, residual peaks were removed and the solvent region was refined as a diffuse contribution without specific atom positions by using the PLATON [21] SQUEEZE module [22] which subtracts electron density from the void regions by appropriately modifying the diffraction intensities of the overall structure. An electron count over the solvent region provided an estimate for the number of solvent molecules removed from the cell. The number of electrons thus located was assigned to seven molecules of water. Applying this procedure led to an improvement in all refinement parameters and minimization of residual peaks.

## 4. Conclusions

A mixture of triterpenoid saponins, known under the collective name escin, which is in current use as a phlebotropic drug, was subjected to consecutive chemical transformations designed and developed as a scalable, validated technical processes, to afford two hitherto unavailable materials: protoescigenin (**1**) and 3,24;16,22-di-*O,O*-isopropylideneprotoescigenin (**2**), in states of high chemical purity. The structures of the compounds was rigorously confirmed by X-ray diffraction and other spectroscopic methods and their specification for medium hydration level (<2.5% of H_2_O) was set according to pharmaceutical intermediate format (SPC/AAN/829-Z for **1** and SPC/AAN/829-Z for **2**). New data on the solid states of the obtained materials are presented, including their solvation, thermal analysis and polymorph examinations. Escin hydrolysis carried out in 3 kg batches affords ca. 300 g of crude protoescigenin **III** or ca. 200 g of pure **1·H_2_O**. Processes are documented as sets of operational instructions, batch reports and validation protocols. Studies on application of **1** and **2** as chemical intermediates will be continued.
